# Liquid Enteral Nutrients Alter the Pharmacokinetics of Orally Administered Carbamazepine in Rats

**DOI:** 10.7150/ijms.71770

**Published:** 2022-04-18

**Authors:** Yoko Urashima, Honami Kobayashi, Kana Yamamoto, Kazuki Matsushita, Kazuya Urashima, Masahiko Tsujikawa, Kaoru Suzuki, Kazumi Kurachi, Masami Nishihara, Masashi Neo, Michiaki Myotoku, Takuro Kobori, Tokio Obata

**Affiliations:** 1 Laboratory of Clinical Pharmaceutics, Faculty of Pharmacy, Osaka Ohtani University, Osaka, Japan.; 2 Department of Pharmacy, Osaka Habikino Medical Center, Osaka, Japan.; 3 Department of Pharmacy, Japan Community Health Care Organization Hoshigaoka Medical Center, Osaka, Japan.; 4 Department of Pharmacy, Japan Community Health Care Organization Osaka Hospital, Osaka, Japan.; 5 Department of Pharmacy, Osaka Medical and Pharmaceutical University Hospital, Osaka, Japan.; 6 Laboratory of Practical Pharmacy and Pharmaceutical Care, Faculty of Pharmacy, Osaka Ohtani University, Osaka, Japan.

**Keywords:** Carbamazepine, enteral nutrient, nasogastric tube, gastrointestinal absorption, antiepileptic drug

## Abstract

The interaction between enteral nutrients (ENs) and drugs co-administered through a nasogastric (NG) tube reportedly affects the absorption and resultant plasma concentrations of the respective drugs. However, the gastrointestinal absorption of carbamazepine (CBZ), an antiepileptic drug, co-administered with liquid ENs through an NG tube has not been clarified. In this study, we measured the recovery rate (%) of CBZ (Tegretol^®^ powder) passed through an NG tube when co-administered with distilled water or ENs (F2α^®^, Racol^®^ NF, Ensure Liquid^®^, and Renalen^®^ LP) of different compositions, frequently used in Japan. We also measured the plasma CBZ level in 26 rats after oral co-administration of CBZ with liquid ENs. The CBZ recovery rate was close to 100% in rats of all EN groups after passage through the NG tube. Furthermore, CBZ area under the plasma concentration-time curve from time zero to 9 h (AUC_0→9h_) of the Ensure liquid^®^ group decreased compared with that of control group (*P* < 0.05) and Renalen^®^ LP group (*P* < 0.01). However, the AUC_0→9h_ of CBZ remained unchanged when co-administered with Ensure liquid^®^ 2 h after initial CBZ administration. In conclusion, the co-administration of CBZ with Ensure Liquid^®^ caused a reduction in the absorption of CBZ from the gastrointestinal tract, without adsorption on the NG tube. The administration of Ensure Liquid^®^ 2 h after CBZ is a way to prevent a decrease in plasma CBZ concentration. Our findings suggest that carefully monitoring the plasma levels of CBZ is necessary in co-administation with Ensure liquid^®^ to prevent the unintended effects of the interaction between CBZ and liquid EN.

## Introduction

Enteral nutrition via a feeding tube directly into the gut is essential for nutritional support in patients who are unable to eat 1. However, the interaction between enteral nutrients (ENs) and drugs, usually co-administered through a nasogastric (NG) tube, has been reported to affect the absorption and resultant plasma concentration of the drugs 2-6. Notably, several studies have reported that the co-administration of ENs alters the blood concentration of phenytoin (PHT), a typical antiepileptic drug, when administered via an NG tube, affecting the drug's ability to suppress epileptic seizures 7-11. Most antiepileptic drugs, including PHT, undergo therapeutic drug monitoring (TDM) because a strictly controlling their levels in the blood is required to ensure efficacy and prevent adverse effects. In our previous study, we had clarified that PHT is not adsorbed on the NG tubes when co-administered with ENs, and the addition of ENs decreased the absorption of PHT in the digestive tract to some extent when orally administered to rats. However, this decrease was not observed when the administration interval between PHT and the ENs was 2 h 12. Therefore, it is important in clinical practice to clarify whether ENs alter the pharmacokinetics of drugs and how to avoid such changes. Hence, more research on the effect of ENs on the adsorption of other antiepileptic drugs to NG tubes and the resultant pharmacokinetics of the target is required.

Carbamazepine (CBZ) is a widely used tricyclic antiepileptic drug and is the first-line drug for treating partial epilepsy; it is frequently used in combination with ENs in Japan. Clark-Schmidt et al. reported that carbamazepine in suspension adsorbs on the NG tube, depending on the dilution method 13. Furthermore, previous studies have reported that semi-solidified ENs and the fiber used for semi-solidification cause a decrease in the gastrointestinal absorption of CBZ after a single oral administration 14-16. However, liquid ENs are currently the most used formulation; thus, it is essential to clarify the interaction between liquid ENs and CBZ. In addition, only Racol^®^ NF among the liquid ENs has been found to not affect the pharmacokinetics of CBZ 14; however, ENs used in clinical practice vary among patients and clinical facilities, and many of them differ in composition from Racol^®^ NF. Moreover, although some fibrous materials have been reported to be carbamazepine adsorbers 15,16, Racol^®^ NF does not contain any fibers, and the effects of co-administration of various fiber-containing liquid ENs on the pharmacokinetics of CBZ are not clear. Therefore, it is necessary to investigate the pharmacokinetics of CBZ following co-administration with various types of liquid ENs.

In this study, we investigated the adsorption of CBZ on the NG tube when co-administered with four types of liquid EN (F2α^®^, Racol^®^ NF, Ensure Liquid^®^, and Renalen^®^ LP; shown in Table 1), including products containing fibers and those frequently used in Japan. We also examined the changes in CBZ pharmacokinetics when CBZ and liquid EN were co-administered orally to rats. Consequently, we found that some liquid ENs alter the pharmacokinetics of CBZ.

## Materials and methods

### Chemicals

CBZ was purchased from FUJIFILM Wako Pure Chemical Co., Ltd. (Tokyo, Japan). Flunitrazepam, as the internal standard, was purchased from Sigma-Aldrich (Tokyo, Japan). Tegretol^®^ powder (2-fold dilution in the presence of a vehicle; Mitsubishi Tanabe Pharma Corporation, Osaka, Japan), F2α^®^ (TERUMO Co. Ltd., Tokyo, Japan), Racol^®^ NF (Otsuka Pharmaceutical Factory, Inc., Tokyo, Japan), Ensure Liquid^®^ (Abbott Japan Co. Ltd., Tokyo, Japan), and Renalen^®^ LP (Meiji Seika Pharma Co., Ltd., Tokyo, Japan) were purchased from commercial sources. For animal anesthesia, sevoflurane was purchased from FUJIFILM Wako Pure Chemical Co., Ltd. Acetonitrile (Nacalai Tesque, Inc., Kyoto, Japan) was of chromatographic reagent grade, and the other chemicals were of analytical reagent grade.

### Passage study of CBZ with EN products through NG tubes

**Figure 1:**
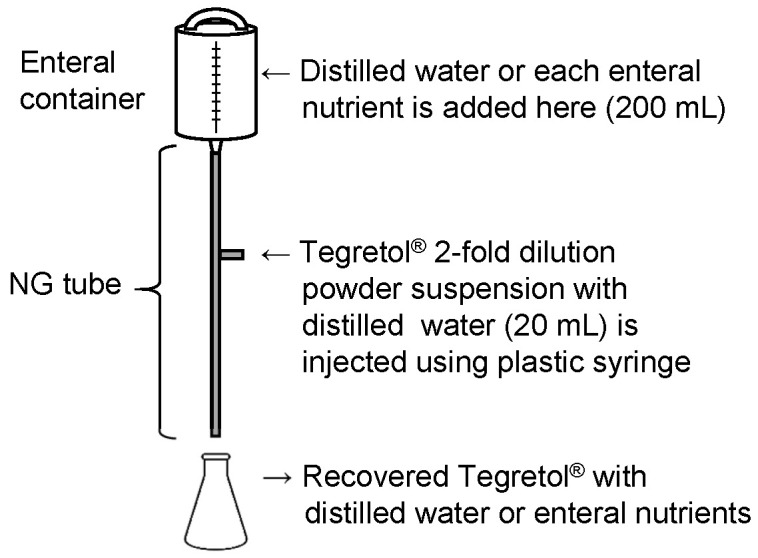
Nasogastric (NG) infusion system for Tegretol^®^ powder with distilled water or enteral nutrients.

First, either 200 mL of each EN (F2α^®^, Racol^®^ NF, Ensure Liquid^®^, or Renalen^®^ LP) or distilled water was added to the enteral administration system. The administration system consisted of an enteral container (Cardinal Health Japan Inc., Tokyo, Japan) and polyvinyl chloride NG tube (inner diameter: 8 Fr, length: 122 cm; NIPRO Co. Ltd., Osaka, Japan) (Fig. 1).

Next, Tegretol^®^ powder (400 mg) in 20 mL of distilled water was administrated using a plastic syringe (NIPRO Co., Ltd., Osaka, Japan) through the side injection port of the catheter. Immediately after the administration of Tegretol^®^ powder suspension, EN was introduced dropwise at a rate of 1-2 mL/min. Finally, the mixture of distilled water or EN and Tegretol^®^ powder suspension was recovered over a period of 2 h from the NG tube outlet into a clean glass flask.

To dissolve Tegretol^®^ powder for facilitating passage through tubes, 400 mL of distilled water was added, and the solution was mixed. Thereafter, 10 mL of this solution was transferred into a 15-mL tube, which was then centrifuged at 3000 rpm for 10 min. Finally, 30 μL of the supernatant was added to 8.97 mL of distilled water in a new tube for neutralization and dilution, and then stored at -30 °C until analysis.

CBZ recovery rate after passage through the NG tube was calculated as follows.

CBZ recovery rate (%) = CBZ (mg) recovered from the outlet of NG tube / injected CBZ (mg) × 100

### Animals

Twenty-six healthy male Sprague-Dawley rats (Japan SLC Inc., Shizuoka, Japan) weighing approximately 200 g (6-7 weeks of age) were used in the oral administration studies. The rats were housed in rooms with a controlled environment (temperature: 22 ± 2 °C, humidity 55% ± 5% under a 12-/12-h light/dark cycle, diurnal time; 0800-2000 h) with food and water provided *ad libitum* for a week. All experimental procedures were conducted in accordance with the Osaka Ohtani University guidelines for the care and use of laboratory animals (approval No. 2004) and the ARRIVE guidelines.

### Oral administration of CBZ co-administered with EN

The rats were cannulated via the right jugular vein under sevoflurane anesthesia 24 h before the drug absorption experiment. The rats were divided into the following six groups: Groups 1-4, co-administered CBZ and one of the four ENs; group 5, administered CBZ only in distilled water (control group); and group 6, Ensure liquid^®^ was administered 2 h after an initial dose of CBZ in distilled water (2h-Ensure liquid^®^ group). CBZ dissolved in water containing 20% (v/v) ethanol and 50% (v/v) propylene glycol (50 mg/kg) 17 was administered orally using a metallic gastric delivery device. Simultaneously, 3 mL/kg EN, F2α^®^, Racol^®^ NF, Ensure Liquid^®^, or Renalen^®^ LP, or distilled water, was administered orally. Plasma samples were collected through the jugular vein cannula at 0.25, 0.5, 1, 2, 3, 5, 7, and 9 h after CBZ solution administration and were replaced with an equal volume of saline. To maintain patency, a small volume of heparinized saline was used to fill the cannula after each blood sample collection. Heparinized saline was removed just before the next blood sample collection, and the rats were sacrificed by deep anesthesia using sevoflurane after collecting the final blood sample. Plasma was obtained by centrifuging the blood at 100 ×*g* for 10 min at 4 °C. The samples were stored at -30 °C until analysis. All efforts were made to minimize suffering to the rats.

### Measurement of CBZ concentration

CBZ concentration in the EN or plasma samples was determined by high-performance liquid chromatography (HPLC). One microgram of the internal standard (flunitrazepam) and 50 μL of acetonitrile were added to 50 µL of the samples. The samples were vortexed for 30 s and centrifuged at 11,200 ×*g* for 20 min at 4 °C. Thereafter, 50 µL of the supernatant was transferred to a clean tube, and 20 µL of the supernatant was injected into the HPLC system.

The HPLC system used was a Shimadzu VP-series consisting of an LC-20AD pump, DGU-20A deaeration unit, CTO-20Avp column oven, SPD-20ADvp UV detector, and SIL-20ADvp auto-injector, controlled by an SCL-20 Avp controller (Shimadzu Co., Kyoto, Japan). Separations were performed on a Cosmosil^®^ 5C_18_-MS-II column (4.6 mm I.D. × 150 mm; Nacalai Tesque, Inc., Kyoto, Japan), preceded by a run through the Cosmosil^®^ 5C_18_-MS-II guard column (4.6 mm I.D. × 10 mm). The mobile phase (15 mM phosphate buffer:acetonitrile (25:55 (v/v)) was flowed at 0.8 mL/min. The column temperature was maintained at 40 °C, and eluting peaks were monitored by UV absorbance at 215 nm.

### Statistical analysis

Pharmacokinetic parameters were calculated using Moment.xls ver. 1.0 18. Statistical analyses were performed using BellCurve for Excel version 2.00 (Social Survey Research Information Co., Ltd., Tokyo, Japan). The results are presented as mean ± standard deviation. The results of each group were analyzed using one-way ANOVA with Tukey's multi comparison test. The threshold for significance was set at* P* < 0.05.

## Results

### Recovery rate of CBZ administered ENs via NG tubes

**Figure 2:**
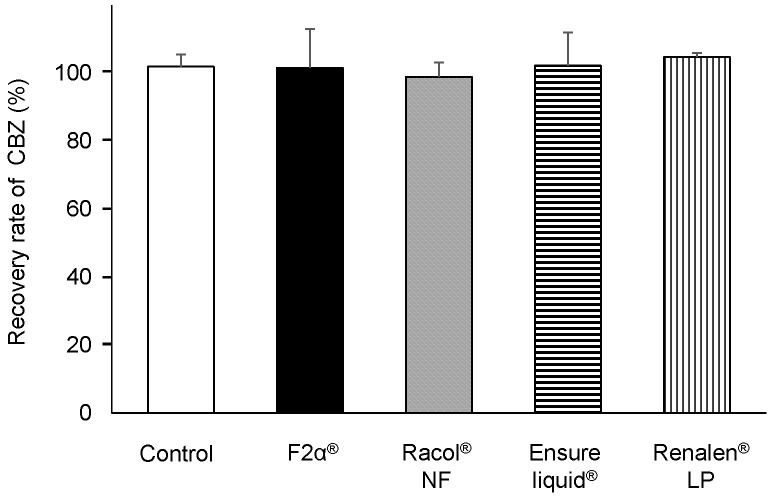
Carbamazepine (CBZ) passage rate through NG tubes with each enteral nutrient. CBZ passage rate through NG tubes with distilled water (control), F2α^®^, Racol^®^ NF, Ensure liquid^®^, and Renalen^®^ LP. Each CBZ passage rate (%) was calculated by dividing the mass of recovered CBZ (mg) from the outlet of the NG tube by the injected mass of CBZ (mg). Data are shown as mean ± S.D. (n = 3).

We prepared a closed enteral administration system consisting of an enteral container and an NG tube for infusion, simulating hospital-ward conditions for EN administration (Fig. 1). CBZ (400 mg) in the form of Tegretol^®^ 2-fold diluted powder, which is widely used in clinics in Japan, was used in these experiments. We measured the amount of CBZ recovered from the outlet of the NG tube 2 h after co-administration with either 200 mL of distilled water (control) or 200 mL of each EN-F2α^®^, Racol^®^ NF, Ensure liquid^®^, or Renalen^®^ LP. The CBZ recovery rate was approximately 100% for all experimental conditions, as well as the control (Fig. 2).

### CBZ pharmacokinetics in rats after oral co-administration with ENs

**Figure 3:**
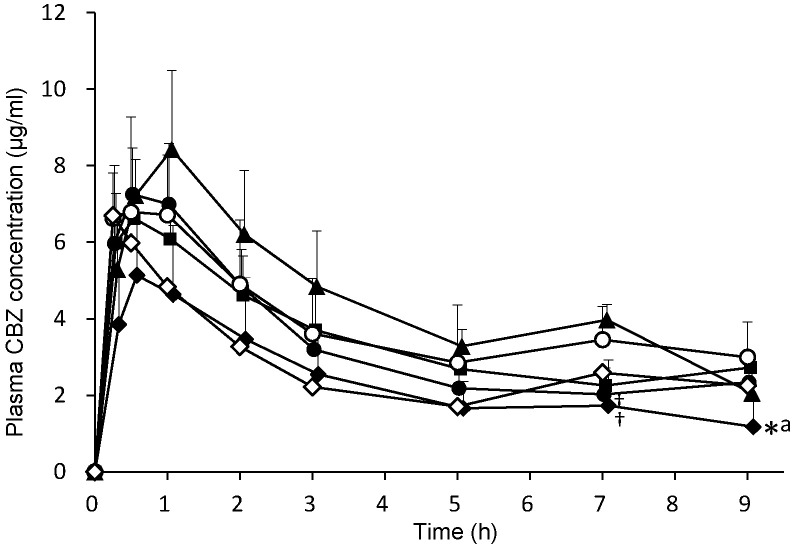
Plasma CBZ concentration profiles after oral administration of each enteral nutrient in rats. CBZ (50 mg/kg) was orally administered with distilled water or each enteral formulation. ○; distilled water (control), ●; F2α^ ®^ group, ■; Racol^®^ NF group, ◆; Ensure liquid^®^ group, ▲; Renalen^®^ LP group, and ◇; Ensure liquid^®^ separate group. Data are shown as mean ± S.D. (n = 4-5). * *P* < 0.05, compared with the control group, ^†^ *P* < 0.05, compared with the Renalen^®^ LP group, ^a^ *P* < 0.05, compared with the Racol^®^ NF group; statistical analyses were performed using Tukey's multiple comparison test.

CBZ (50 mg/kg) combined with 3 mL/kg distilled water (control group) or each EN (F2α^®^, Racol^®^ NF, Ensure liquid^®^, Renalen^®^ LP and 2h-Ensure liquid^®^) was orally administered to rats. Plasma CBZ concentrations at 0.25, 0.5, 1, 2, 3, 5, 7, and 9 h after administration are shown in Fig. 3. Plasma CBZ concentration 7 h after co-administration with F2α^®^ and Ensure liquid^®^ significantly decreased compared with that of the Renalen^®^ LP group, and the plasma CBZ concentration 9 h after co-administration with Ensure liquid^®^ significantly decreased compared with that of the control group.

The pharmacokinetic parameters of CBZ are presented in Table 2. CBZ area under the plasma concentration-time curve from time zero to 9 h (AUC_0→9h_) of the Ensure liquid^®^ group significantly decreased compared with that of the control group (*P* < 0.05) and Renalen^®^ LP group (*P* < 0.01). There was no significant difference in the parameters of maximum plasma concentration (C_max_), time to reach C_max_ (T_max_), and mean residence time from time zero to 9 (MRT_0→9h_) among all EN groups compared with each other.

CBZ (50 mg/kg) was orally administered with distilled water or each enteral formulation-distilled water (control), F2α^®^, Racol^®^ NF, Ensure liquid^®^, Renalen^®^ LP, and Ensure liquid^®^ groups. AUC_0→9h_, area under the concentration-time curve from time zero to 9 h; C_max_, plasma maximum concentration; T_max_, time to reach peak serum concentration; MRT_0→9h_, mean residence time were measured. Data are shown as mean ± SD (n = 4-5). The symbol (*) denotes *P* < 0.05, compared with the control group and (^††^) denotes *P* < 0.01, compared with the Renalen**^®^** LP group; statistical analyses were performed using Tukey's multiple comparison test.

## Discussion

First, we investigated CBZ adsorption on the NG tubes with ENs, and clarified that CBZ did not adsorb on the commonly used 8 Fr caliber NG tubes, indicating that the entire dose of the orally administered CBZ reached the gastrointestinal tract (Fig. 2). Clark-Schmidt et al. reported that undiluted CBZ suspension adsorbed on the NG tube^13^, but 50% diluted solution with water did not; thus, dilution of the CBZ suspension with EN may have prevented the adsorption of CBZ on the NG tubes in the present study.

The co-administration of Ensure liquid^®^ decreased the plasma CBZ concentration and the AUC_0→9h_ compared with the co-administration of distilled water. Interestingly, there were no significant changes observed when Ensure liquid^®^ was administered separately. These results indicate that the gastric absorption amount of CBZ co-administered with Ensure liquid^®^ decreased and that the administration of Ensure liquid^®^ 2 h after CBZ is an effective approach for preventing any decrease in plasma CBZ concentration. These findings suggest that careful monitoring of CBZ plasma level is necessary in clinical practice while co-administering the drug with Ensure liquid^®^ to prevent the interaction between CBZ and liquid EN. Ohnishi et al. also showed that oral co-administration of Sho-saiko-to extract powder and CBZ reduced the gastrointestinal absorption of CBZ, at least partially, by delaying gastric emptying without affecting CBZ metabolism 17, and Singh et al. reported that the oral administration of 10 g/kg soybean decreased the absorption of CBZ and suppressed gastric cavitation in rats 19. These findings indicate that decreased gastric emptying rate (GER) is a factor in decreased absorption of CBZ. Moreover, it has been reported that Ensure liquid^®^ tended to reduce GER compared with distilled water and Racol^®^ NF. Therefore, we hypothesized that CBZ absorption changed in this study because Ensure liquid^®^ affected the GER. In addition, there is a possibility that decreased absorption of CBZ co-administered with Ensure liquid^®^ may be caused by CBZ binding to the contents in Ensure liquid^®^. This warrants further investigation in this regard.

F2α^®^ and Renalen^®^ LP, which contain fiber, did not reduce the AUC of CBZ administered orally. These results suggest that liquid EN containing fiber does not necessarily affect the gastric absorption of CBZ. F2α contains 2% guar gum degradation products; this concentration is higher than 1% guar gum reported to have affected the adsorption of CBZ 16. However, the guar gum degradation product is less viscous than guar gum, which may have prevented it from affecting the blood concentration of CBZ. Indigestible dextrin contained in Renalen^®^ LP is the fiber reported to inhibit the absorption of sugar and fat from the digestive tract 20,21. The interaction with drugs and indigestible dextrin has not been clarified, but this study showed that indigestible dextrin may not affect the gastric absorption of CBZ. Our findings indicate that the effect of liquid EN on CBZ pharmacokinetics needs to be evaluated comprehensively including not only fibers but also other components. In addition, our data demonstrated that liquid Racol^®^ NF did not affect the pharmacokinetics of CBZ, which is consistent with the result of Nagai et al. 14.

Furthermore, we previously reported that the gastric absorption of PHT, co-administered with F2α^®^ and Racol^®^ NF, containing a large amount of protein components (Table 2), significantly decreased, and these ENs are different from those used in the present study. Therefore, we speculate that binding to the protein components in ENs decreases PHT absorption in the gastrointestinal tract because of the high rate of plasma protein binding. On the contrary, CBZ has a lower rate of plasma protein binding than PHT; thus, F2α^®^ and Racol^®^ NF may not have caused the reduction in CBZ gastric absorption.

Co-administration of Renalen^®^ LP increased the AUC of CBZ, which increased the gastric absorption of CBZ. The osmotic pressure of Renalen^®^ LP is 720 mOsm/L, which is significantly higher than that of other ENs, at 300-360 mOsm/L. Funai et al reported that apple juice, the osmotic pressure of which is approximately 750 mOsm/L, promotes water secretion in the rat intestine 22. We hypothesized that the solubility of CBZ, which is poorly soluble, was increased by the secretion of water by Renalen^®^ L in the gastrointestinal tract, resulting in increased CBZ absorption. The administration of Ensure liquid^®^ 2 h after CBZ prevented the decrease in plasma CBZ concentration in co-administration of water, however it could not prevent in co-administration of Renalen^®^ LP. This result indicates that caution should be exercised when changing the type of ENs.

This study had a limitation as the GER with EN and long-term EN administration were not examined. Further investigation is needed to determine if the administration of liquid EN actually alters the GER. Moreover, several studies have reported that the long-term administration of nutritional components affects CBZ metabolism. It has been reported that CYP3A4, the main metabolic enzyme of CBZ, is induced by the repeated administration of soybean 19 and honey 23. Notably, the EN used in this study contained soybean protein and soy isoflavone, similar to flavonoids in honey; thus, their long-term administration may affect the metabolism of CBZ. Further investigations of CBZ concentration profiles with repeated EN administration are needed.

## Conclusion

In this study, we clarified that CBZ co-administered with F2α^®^, Racol^®^ NF, Ensure Liquid^®^, and Renalen^®^ LP completely passed through the NG tube and reached the gastrointestinal tract. Furthermore, Ensure Liquid^®^ decreased the gastric absorption of CBZ, and this interaction could be avoided by first administering CBZ, waiting for 2 h, and then administering Ensure Liquid^®^. Our findings suggest that carefully monitoring the plasma levels of CBZ is necessary in co-administation with Ensure liquid^®^ to prevent the unintended effects of the interaction between CBZ and liquid EN.

## Figures and Tables

**Table 1:** List of main components per 200 mL of each enteral nutrient

		F2α^®^	Racol^®^ NF	Ensure liquid^®^	Renalen^®^ LP
Protein	Total amount (g)	10	8.8	7	3.2
	Composition (g)	Milk protein (8.0)	Milk casein (7.8)	Casein sodium (6.8)	Milk protein (UNK)
		Soy protein (2.0)	Soy protein isolate (3.4)	Soy protein isolate (1.0)	
Carbohydrate	Total amount (g)	30.2	31.2	27.4	59.2
	Composition (g)	Dextrin (UNK)	Maltodextrin (29.8)	Dextrin (19.6)	Dextrin (UNK)
		Sucrose (UNK)	Sucrose (2.6)	Sucrose (8)	Sucrose (UNK)
Lipid	Total amount (g)	4.4	4.4	7	9
	Composition (g)	Medium-chain triglyceride (UNK)			Medium-chain triglyceride (UNK)
		Soybean oil (UNK)	Soybean oil (1.4)	Soy Lecithin (0.32)	
		Canola oil (UNK)			Canola oil (UNK)
			Tricaprylin (1.5)		
			Perilla oil (0.36)		
			Palm oil (0.66)		Palm oil (UNK)
				Corn oil (6.6)	
					Fish oil (UNK)
	Medium-chain: Long-chain triglyceride	1:1	Long-chain triglyceride only	Long-chain triglyceride only	1:4
Fiber (g)		Guar gum degradation (4.0)	―	―	Indigestible dextrin (3.2)
Osmotic pressure (mOsm/L)		370	300-360	330	720
pH		7.0	6.0-7.2	6.6	6.2
Calorie (kcal)		200	200	200	320
Viscosity (mPa . s )		10.0	5.5-6.5	9.0	15.0
Specific gravity (g/cm^3^)		1.08	1.08	1.10	1.12

UNK; unknown.

**Table 2:** Pharmacokinetic parameters of carbamazepin after oral administration with each enteral formulations in rats

	Control	F2α®	Racol® NF	Ensure liquid®	Renalen® LP	Ensure liquid® separate
AUC_0→9h_ (µg . h/mL)	35.34±8.00	29.85±3.55	31.56±4.62	21.61±8.47 * ††	40.39±6.32	27.19±6.24†
Cmax (µg/mL)	7.73±1.22	7.63±1.31	6.85±0.57	5.15±1.37	8.49±1.96	6.82±0.90
T_max_ (h)	0.69±0.38	0.70±0.27	0.56±0.31	0.60±0.22	0.88±0.25	0.31±0.13
MRT_0→9h_ (h)	3.88±0.40	3.43±0.36	3.64±0.11	3.34±0.57	3.71±0.18	4.10±0.44

CBZ (50 mg/kg) was orally administered with distilled water or each enteral formulation: distilled water (control), F2α^®^, Racol^®^ NF, Ensure liquid^®^, Renalen^®^ LP, and 2h-Ensure liquid^®^ groups. AUC_0-9h_, area under the concentration-time curve from time zero to 9 h; C_max_, plasma maximum concentration; T_max_, time to reach peak serum concentration; MRT_0-9h_, mean residence time. Data are shown as mean ± SD (n = 4-5). * *P* < 0.05, compared with control group and ^††^ *P* < 0.01 or ^†^ *P* < 0.05 compared with Renalen**^®^** LP group; statistical analyses were performed using Tukey's multiple comparison test.

## Abbreviations

AUC_0→9h_: area under the plasma concentration-time curve from time zero to 9 h
CBZ: carbamazepine
C_max_: maximum plasma concentration
EN: enteral nutrient
HPLC: high-performance liquid chromatography
MRT_0→9h_: mean residence time from time zero to 9
NG: nasogastric
PHT: phenytoin
TDM: therapeutic drug monitoring
T_max_: time to reach C_max_
